# Increasing temperature denatures canine IgG reducing its ability to inhibit heartworm antigen detection

**DOI:** 10.1186/s13071-023-05739-8

**Published:** 2023-04-28

**Authors:** Jeff M. Gruntmeir, Jeff R. Abbott, Peter E. Kima, Maureen T. Long, Byron L. Blagburn, Heather S. Walden

**Affiliations:** 1 Emerging Pathogens Institute, University of Florida, Gainesville, FL USA; 2 Department of Infectious Diseases & Immunology, College of Veterinary Medicine, University of Florida, Gainesville, FL USA; 3 Southeastern Center of Excellence in Vector Borne Diseases, Gainesville, FL USA; 4 Department of Comparative, Diagnostic, and Population Medicine, College of Veterinary Medicine, University of Florida, Gainesville, FL USA; 5 Department of Microbial and Cell Science, College of Veterinary Medicine, University of Florida, Gainesville, FL USA; 6 Institue for Food and Agricultural Sciences, College of Veterinary Medicine, University of Florida, Gainesville, FL USA; 7 Washington State University, Pullman, WA USA; 8 Auburn University, Auburn, AL USA

**Keywords:** Immune complex dissociation, Heat treatment, Antigen, Antibody, Dirofilaria immitis, Canine heartworm, Linear Epitope, Immunodiagnosis, Immune complex, Heartworm

## Abstract

**Background:**

Immune complexing of target antigen to high affinity host antibody is recognized to impact the sensitivity of commercial heartworm antigen tests. Published information describing the effect of heat on interfering canine host antibodies is lacking. Immune complex dissociation (ICD) by heat treatment of serum for samples initially testing negative for heartworm antigen increases sensitivity of commercial antigen tests, particularly for single sex or low adult infection intensities. In this study the stability and nature of the targeted epitope and mechanism of heat ICD were examined.

**Methods:**

Canine IgG was isolated using protein-A columns from serum originating from four dogs evaluated after necropsy: one dog with evidence of previously cleared infection and three dogs with confirmed heartworm infections. These dogs were expected to have an excess of antibodies based on negative antigen test and to have no or low antigen optical density, respectively, following heat treatment. Interference of antigen detection on (non-heated) positive serum was evaluated, following 1:1 mixing of antibody/PBS solutions previously heated at 25 °C, 65 °C, 75 °C, 85 °C, 95 °C and 104 °C, compared to positive serum/PBS control measured by optical density using a commercial heartworm antigen ELISA and protein quantification. Live heartworms incubated in media for 72 h provided excretory/secretory antigen for antigen stability studies following heat, endopeptidase digestion and disulfide bond reduction.

**Results:**

Mixing antigen-positive heartworm serum with antibody solutions demonstrated a significant inhibition of antigen detection for antibody solutions previously heated at 25 °C and 65 °C relative to positive serum/PBS control. Antigen detection optical density was restored at or above the control when positive serum was mixed with solutions previously heated at 75 °C, 85 °C, 95 °C and 104 °C. Significant changes occurred in protein levels for antibody solutions heated at 75 °C, 85 °C, 95 °C and 104 °C. Relative stability of antigen from live heartworms in culture was demonstrated following heat, chemical and enzymatic treatment.

**Conclusions:**

Significant changes in protein levels and antigen binding ability occurred in IgG solutions heated above 65 °C. The findings confirm heat denaturation of antibodies as the suspected mechanism of heat ICD at 104 °C for antigen diagnosis of heartworm. No significant change occurred in antigen detection following heat, chemical or enzymatic digestions supporting a heat-stable linear nature of the epitope.

**Graphical Abstract:**

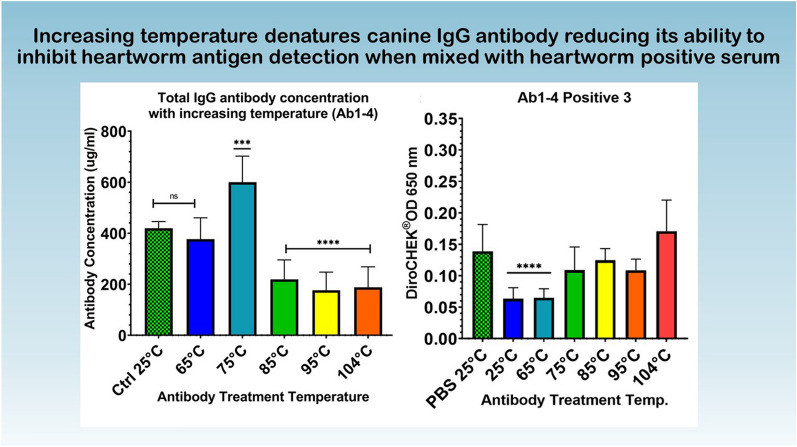

## Background

Immune complexes of antibody and antigen have been historically recognized as a factor influencing serological detection of filarial infections including the canine heartworm, *Dirofilaria immitis* [1–4]. Initial investigation of direct serological detection of amicrofilaremic filariasis focused on precipitation of immune complexes and detection following a secondary method for dissociating antigen from antibody, generally referred to as immune complex dissociation (ICD), by chemical or heat treatment of serum [1–4]. The use of ICD protocols has previously been considered necessary to improve the sensitivity for detection of antibody or antigen targets resulting from viral, fungal, protozoal and other infectious organisms [5–8]. Historically, heat as an (ICD) step was included in heartworm antigen protocols from 1985 to 1994 and currently remains in some reference laboratory in-house tests [8, 9]. Additionally, heat is often used in immunohistochemistry to reveal target epitopes [10]. Despite these applications, little published information exists on the mechanism for which heat decreases binding of host antibodies that may interfere with detection of target heartworm antigen.

The diagnostic practice of heat treatment (heat ICD) of serum at 104 °C prior to antigen testing has demonstrated an increased heartworm antigen detection when clinical suspicion of infection is suspected, despite an initial negative antigen test [8, 9, 11–16]. When applied to subcutaneously induced experimental heartworm infections in dogs (*n* = 12), the use of heat ICD improved time to initial antigen detection to 98–142 days from 140 to 217 days for heated and non-heated serum, respectively [16]. In two recent studies, heat ICD increased sensitivity by 7.7% and 19.6% for mature heartworm infections including infections of low numbers of mixed or single sex using sera from necropsy-verified natural infections [9, 16]. This increased sensitivity is suggested to result from denaturation of antibodies bound to the target heartworm antigen and potentially concentration of the antigen due to the reduced volume of supernatant post-heat ICD [7–9, 11–17].

Immune complexing causing false-negative results on heartworm tests is likely due to an excess of antibodies to the target antigen, binding to epitopes also targeted by heartworm antigen test reagents [8, 11]. An excess of antibodies may also be induced, resulting in a false-negative antigen test, following initiation of macrocyclic lactones [14] as part of a standalone non-arsenical treatment of moxidectin and doxycycline [18] or as part of a treatment protocol in the months prior to melarsomine injections [19], the only approved heartworm treatment [14, 18, 19]. Following heat ICD, the targeted glycoprotein antigen is suggested to remain soluble in the supernatant portion of the sample following centrifugation, potentially concentrated because of a decrease in sample volume [15] and likely uninhibited by denatured antibody [8, 11]. Previously reported instances of large increases in measured optical density were observed following heat ICD, aside from increased antigen concentration, likewise also indicating high titers of antibody relative to antigen [7, 11, 16]. Unpublished data, using samples from previous studies [16] where antigen was initially undetectable but seroconverted to post-heat ICD antigen positive with large increases in OD will seroconvert back to being undetectable when mixed back 1:1 with the same samples’ original nonheated portion of serum. This suggests a heat-stable antigen present in the post-heat ICD supernatant remains in a form susceptible to immune complexing   likely due to an epitope unaffected by possible denaturation and still recognized by Fab regions on non-heated canine antibody. Although information is lacking regarding impacts of heat on the canine IgG antibody, characterizing heat's effect over a range of temperatures may be informative for diagnostic strategies for heartworm or other infections with diagnostic targets prone to immune complexing. Additionally, little information on the stability of the targeted heartworm antigen or nature of the epitope recognized by host antibody and commercial test reagents has been reported outside the original published articles on the development of heartworm antigen detection [2–4].

The primary purpose of this research was to address questions regarding the mechanism of heat ICD in relation to heartworm antigen detection by testing two hypotheses: (i) that increased heat improves heartworm antigen detection resulting from heat-induced changes to the heartworm antigen-specific antibody and (ii) that increased heat does not increase heartworm antigen detection due to changes in the target antigen’s protein conformation or nature of the targeted epitope.

## Methods

### Isolation of canine immunoglobulin G (IgG)

Archived canine serum samples collected for a previous study were selected for antibody isolation based on an initial negative antigen result (DiroCHEK^®^, Zoetis, Kalamazoo, MI) and either a low post-heated serum antigen result [optical density (OD) 650 nm] (14) for necropsy-positive samples or a negative post-heated serum antigen result for the one sample with only embolized worm fragments at necropsy [9]. Candidate samples meeting the above criteria underwent antibody isolation from 1.5 ml serum each using Protein A spin columns (nAb™ Protein A Plus Spin Kit, Thermofisher™). Antibodies eluted were resuspended to 2× concentration of initial serum volume in phosphate-buffered saline (PBS), pH 7.2. Antibody solutions obtained from these samples were tested on the DiroCHEK^®^ both before and after heat ICD at 104 °C to ensure no detectable antigen carryover in isolated antibody would impact downstream experiments. Antibody solutions from four dogs were determined free of detectable antigen: one dog with evidence of previously cleared infection based on visible pulmonary pathology with embolized worm fragments and three dogs with confirmed heartworm infections at necropsy. Details of heartworm infection status for these four samples are listed in Table 1. Additional antibody was isolated by Protein A columns from serum of these four dogs using a total of 7 ml serum from each, according to the manufacturer’s procedures. All recovered antibody elutants obtained were pooled for each animal, concentrated and washed with 6 ml PBS (Vivaspin 2, 100 kDa MWCO protein concentrator, GE Healthcare), recovered and resuspend in 3.5 ml PBS.

**Table 1:** Heartworm status and information on samples used for canine antibody isolation and nonheated positive serum

Antibody solutions	Sample ID	Heartworm infection status	Pre-/post-heated serum antigen	Heartworm positive samples	Sample ID	Heartworm infection status
Ab1	19013	3 Male	Negative/positive	Ag1	18004	2 Male 1 Female
Ab2	19006	Embolized fragments	Negative/negative	Ag2	18006	1 Male 1 Female
Ab3	19002	4 Female Immature	Negative/positive	Ag3	18035	4 Male 2 Female
Ab4	18075	3 Male	Negative/positive	Ag4	18046	1 Male 2 Female

### Changes in protein concentration of IgG antibody solutions with increasing temperature

For each of the four antibody solutions (Ab1–4) evaluated, six 330-µl aliquots were prepared in individual 1.5 microcentrifuge tubes. Individual aliquots of each Ab1–4 (6 for each, 24 total) were heated in a dry heat block each at six temperatures: 25 °C, 65 °C, 75 °C, 85 °C, 95 °C and 104 °C for 10 min. Heated antibody aliquots were immediately centrifuged at 16000×*g*, followed by manual disruption of protein precipitant using a pipet tip and centrifugation at 16000×*g* for 10 min; recovered supernatant was adjusted back to initial total volume of 330 µl with sterilized, ultrapure, 18.2 Ohm deionized water. Heated antibody aliquots from Ab1–4 had total protein concentrations measured by fluorometer (Qubit™ 2.0, Invitrogen™) using the Qubit™ Protein Assay Kit (Q33211) according to the manufacturer’s instructions.

### Effect of increasing temperature on IgG antibody inhibition of antigen detection

To investigate the hypothesis that increased heat improves heartworm antigen detection resulting from heat-induced changes to the antigen-specific antibody, we measured changes in total protein concentration of Ab1–4 following heating at 6 temperatures and evaluated interference of antigen detection/OD of (nonheated) positive serum following 1:1 mixing with these previously heated canine IgG antibody solutions compared to the OD of positive serum mixed with PBS control.

Antigen-positive samples were obtained from dogs determined to be heartworm positive at necropsy and antigen positive without heat ICD (Ag1–4) were used to mix with each heated antibody aliquot described above. Each heated aliquot (described above) from Ab1–4 was mixed 1:1 with three aliquots of each antigen-positive sample Ag1–4 (80 µl non-heated serum into each tube with 80 µl heated antibody solution), mixed by vortex and allowed to sit 5 min at room temperature. Samples were again mixed by vortex and tested in triplicate by the DiroCHEK^®^ and OD read at 650 nm (Synergy HTX Multi-Mode Microplate Reader, Biotek Instruments, Inc., Winooski, VT) [14]. The wavelength for reading optical density at 650 nm used in this study previously originated from the antigen test’s “lab protocol,” previously included and since removed (~2015) from the manufacturer’s package insert. Since aliquots of Ab1–4 in PBS were mixed 1:1 with the antigen-positive samples, each antigen-positive sample was also diluted 1:1 with PBS alone and included as the appropriate control for comparison. Graphing and statistical analysis were completed using Graphpad Prism 9.1.2 using one-way ANOVA and Dunnett correction for multiple comparisons.

### Recovery and maintenance of live adult *D. immitis* in media for recovery of excretory and secretory antigens

To collect *D. immitis* excretory/secretory crude antigen (ES antigen) free of antibody, live heartworms recovered as part of previous studies were used [9]. Altogether, nine sets of recovered mature heartworms consisting of three groups of two males each, three groups of two females each and three groups of two males/two females each were briefly rinsed in warm tap water to lyse and remove blood clots and visually assessed to determine sex, viability and length measured to confirm maturity [20, 21]. The nine groups of mature heartworms were briefly transferred to individual sterile 50-ml centrifuge tubes containing 40 ml sterile water and gently inverted three times. The worms were then transferred sequentially to new sterile tubes with 40 ml sterile PBS and inverted three times, then to 40 ml PBS supplemented with antibiotic/antimycotic (1× penicillin/streptomycin/amphotericin B). All tubes were pre-warmed to 37 °C, and heartworms were incubated in the final solution for 30 min. Each group of washed heartworms was transferred to 25 cm^2^ sterile vented cap culture flasks containing 50 ml pre-warmed antibiotic/antimycotic supplemented media (RPMI 1640 with l-Glutamine and 25 mM HEPES). Vented flasks were incubated upright for 72 h at 37 °C with 5% CO_2_. Heartworm viability was checked visibly for active movement during media collection at 24, 48 and 72 h. The media were replaced at 24 and 48 h. Collected media containing ES were filtered to remove any released microfilariae using 5.0-µm filters and further filtered through a sterile 0.45-µm filter. Culture supernatant recovered over the 72 h was pooled for each of the nine groups of cultured heartworms and used for further experiments. All heartworm ES antigen solutions were maintained at 4 °C until use. All heartworms transferred to flasks and incubated remained viable at 72 h. Details of heartworm infection status for these four positive samples are listed in Table 1.

### Effect of temperature on cultured heartworm ES Media Antigen (ES media) measured by protein concentration and antigen detection

To investigate the hypothesis that increased heat does not increase heartworm antigen detection because of changes in the target antigen’s protein conformation, we measured total protein and antigen detection (OD 650 nm) as an indicator of a potential change in conformation following heating of excretory/secretory antigens recovered in media (ES media) at increasing temperature increments. Six aliquots (160 µl each) of each pooled ES medium were heated in a dry heat block for 10 min using one aliquot for each of six temperatures (25 °C, 65 °C, 75 °C, 85 °C, 95 °C and 104 °C) and immediately centrifuged at 16000×*g* for 10 min. Total protein was measured for each of the six heated aliquots (six aliquots per each of the nine pooled E/S media solutions) by fluorometer (Qubit™ 2.0, Invitrogen)™ using the Qubit™ Protein Assay Kit (Q33211), according to the manufacturer’s instructions. Graphing and statistical analysis were completed using Graphpad Prism 9.1.2 using two-way ANOVA and Dunnett correction for multiple comparisons. These previously heated ES aliquots were additionally tested in triplicate by DiroCHEK^®^ with results determined by measuring the OD 650 nm. Graphing and statistical analysis were completed using Graphpad Prism 9.1.2 using two-way ANOVA and Dunnett correction for multiple comparisons.

### Endopeptidase enzymatic treatment by immobilized pepsin and disulfide bond reduction by TCEP of ES media

To further investigate the linear or conformational nature of the target antigen’s epitope, we measured total protein and antigen detection (OD 650 nm) following endopeptidase digestion and disulfide bond reduction of aliquots from each of the nine pooled heartworm ES media. Briefly, 200 µl aliquots of each ES media culture in 1.5 ml microcentrifuge tubes were mixed with pepsin, a nonspecific endopeptidase immobilized on beaded agarose resin (Pierce™ Thermofisher Scientific™ #20343), and digested for 4 h with constant shaking at 37 °C, according to the manufacturers’ directions. Following centrifugal separation of resin with immobilized pepsin from digested ES antigen, supernatant aliquots were tested in triplicate by the DiroCHEK^®^ with results determined by measuring the OD 650 nm.

As another way to determine whether the targeted antigen epitope may have a conformational aspect contributed by disulfide bonding between peptide chains, these bond types were reduced and tested for changes in antigen detection measured by OD. For each of nine pooled heartworm ES antigens evaluated, 200 µl aliquots of each ES media culture in 1.5 ml microcentrifuge tubes were mixed with Tris[2-carboxyethyl] phosphine hydrochloride (TCEP), a trialkylphosphine disulfide bond reducing agent, immobilized on beaded agarose gel (Pierce™ Thermofisher Scientific™ #77712) and incubated at room temperature for 30 min with constant shaking, according to the manufacturer's directions. Following the incubation period and centrifugal separation of immobilized TCEP from disulfide bond reduced ES antigen, the supernatant aliquots were immediately tested in triplicate by the DiroCHEK^®^ with results determined by measuring the OD 650 nm.

## Results

### Effect of increasing temperature on canine IgG antibody interference of heartworm antigen detection as measured by total protein and antigen detection measured by optical density (OD)

**Fig. 1:**
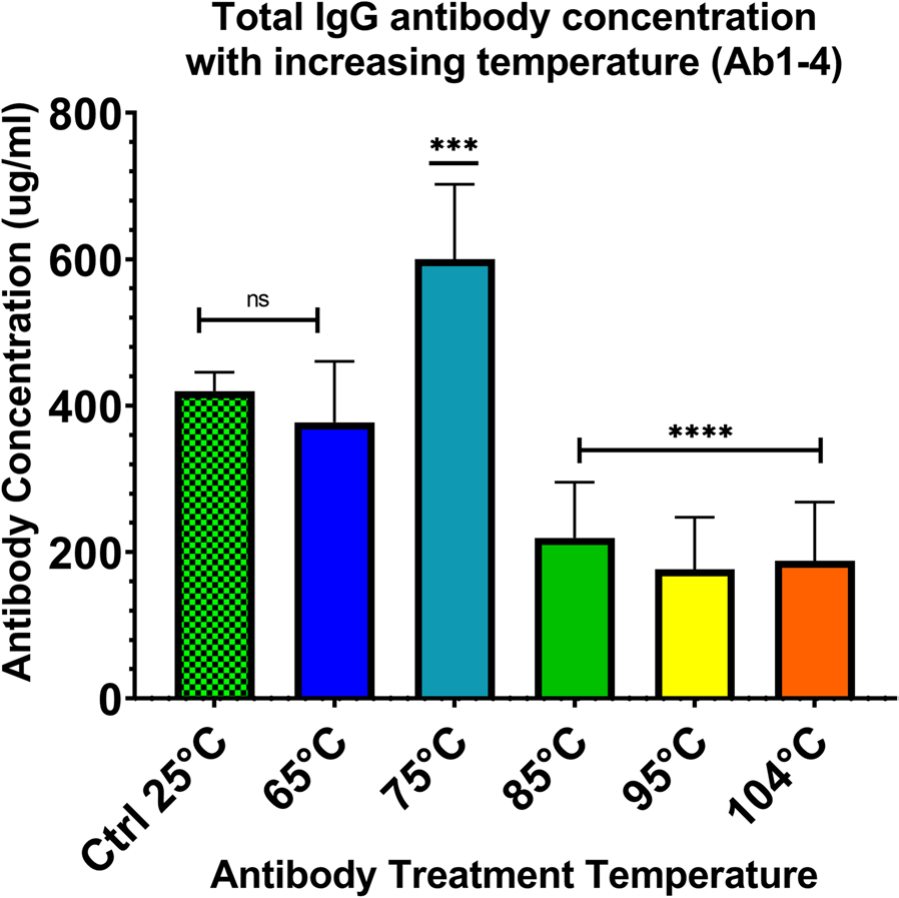
Change in IgG antibody concentration following heating of antibody solutions (Ab1–4) at six temperatures as measured by total protein. Protein concentrations were significantly increased when heated at 75 °C and were significantly decreased for antibody solutions heated at 85 °C, 95 °C and 104 °C

Relative to the 25 °C control temperature, no significant change in protein concentration was seen following heating at 65 °C. A significant increase in protein concentration was observed at 75 °C and significant decreases in protein concentration observed for aliquots heated at 85 °C, 95 °C and 104 °C, all relative to the 25 °C control temperature. Results for protein concentration measurements of heated antibody aliquots adjusted to original starting volume are displayed in Fig. 1.

**Fig. 2:**
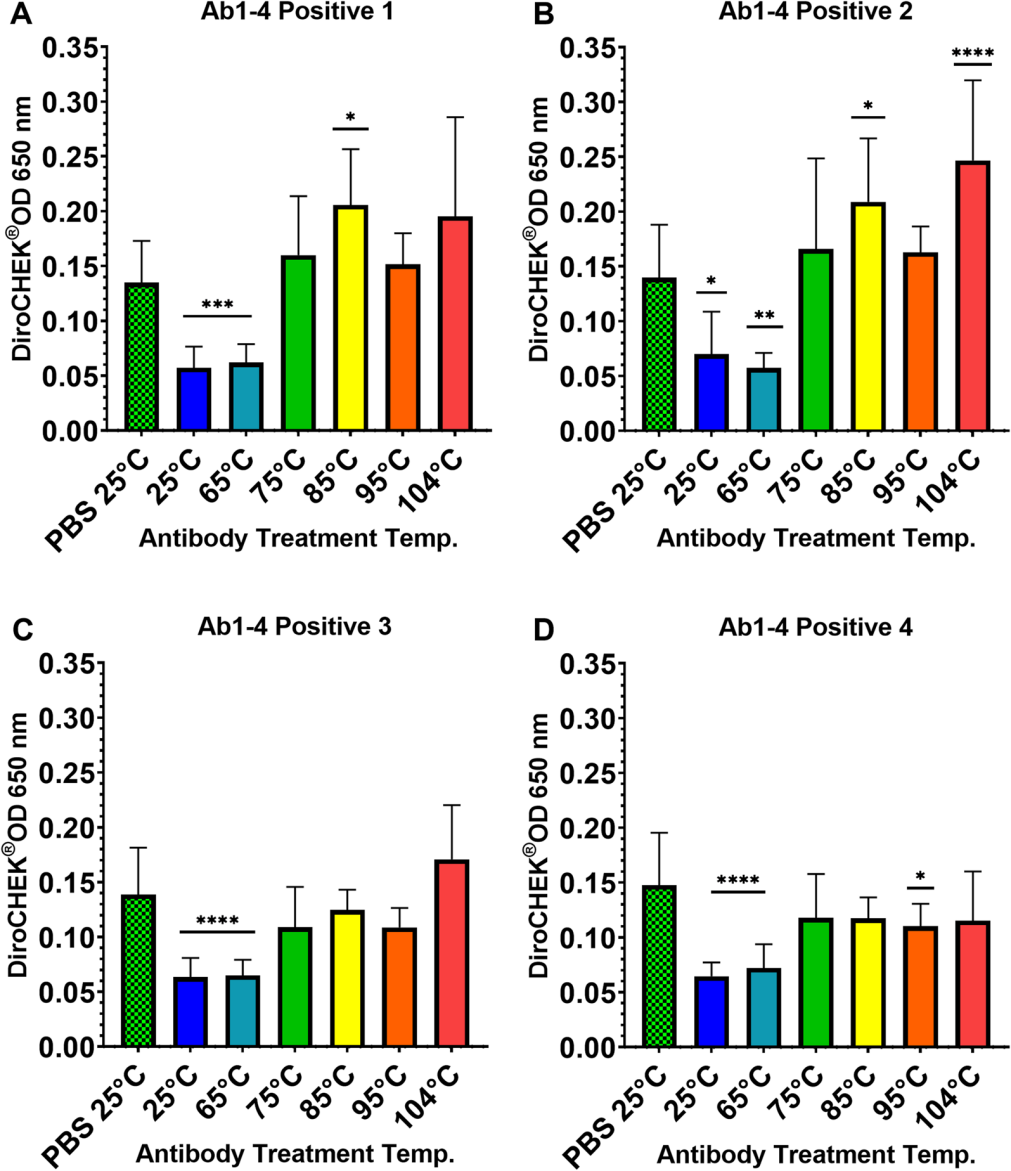
Effect of increasing temperature on canine IgG antibody interference of antigen detection as measured by DiroCHEK^®^ via optical density at 650 nm. Pooled heartworm antigen optical density results following mixing of antibody solutions Ab1–4 (heated at 6 temperatures) with four heartworm antigen positive samples Ag1–4 (Graphs A-D) compared to Ag1–4/PBS 25 °C controls. Antigen detection is initially decreased following mixing with antibody solutions and then recovers with increasing temperature relative to PBS control

Antigen detection among the four antigen-positive heartworm samples Ag1–4 following mixing with both the 25 °C and 65 °C heated antibody/PBS solutions showed significant decreases in antigen detection (measured by OD 650 nm) compared to OD of Ag1–4 mixed with PBS control (free of added antibody). Antigen detection/OD detected in Ag1–4 following mixing with the 75 °C, 85 °C, 95 °C and 104 °C heated antibody solutions showed non-significant differences or significant increases compared to OD of respective Ag1–4 mixed with PBS control. Results measuring antigen detection in positive samples mixed with either PBS control or heated antibody aliquots are displayed in Fig. 2.

### The effect of temperature on protein concentration and antigen detection of cultured heartworm ES media

**Fig. 3:**
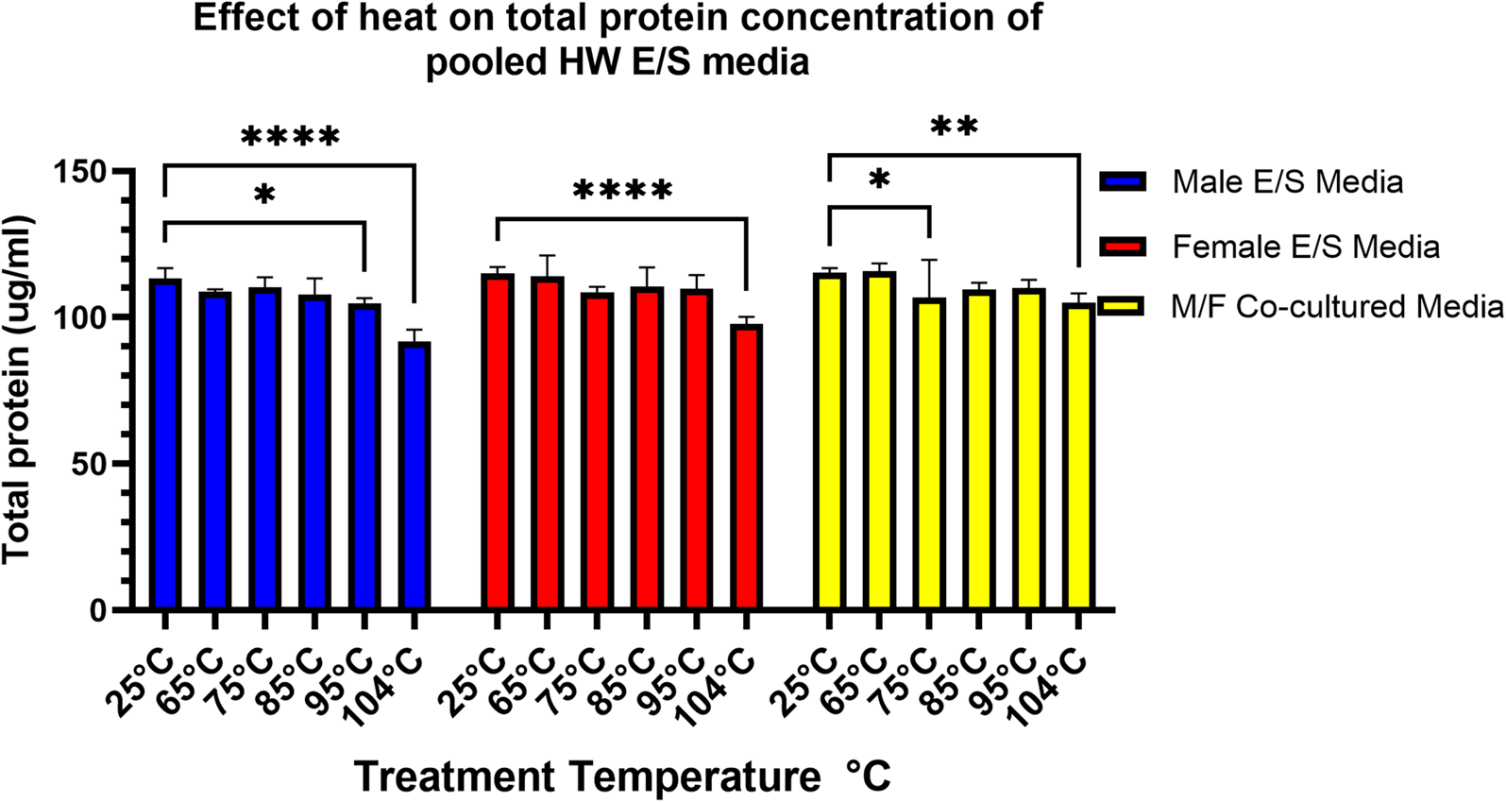
Change in protein concentration with increasing temperature for pooled excretory/secretory (E/S) media from live heartworms. Protein concentrations represent total protein, and changes may not reflect changes to target heartworm antigen

**Fig. 4:**
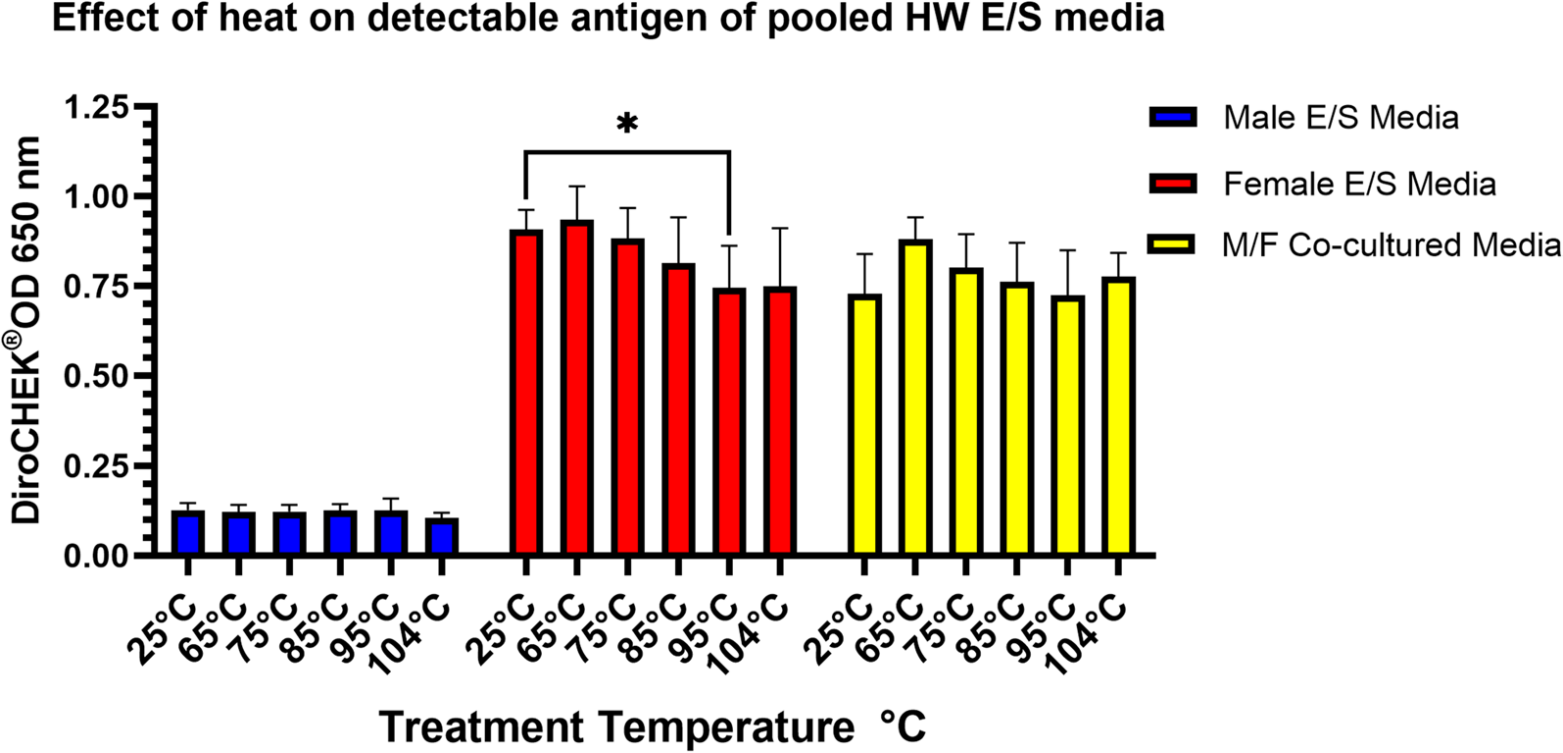
Effect of increasing temperature on antigen detection in pooled excretory/secretory (E/S) media from live heartworms as measured by DiroCHEK^®^ via optical density at 650 nm

The total protein levels for ES media remained relatively unchanged with increasing temperature (Fig. 3), although significant differences were noted at 95 °C and 104 °C for male E/S media and at 104 °C for both female and co-cultured E/S media. No visible precipitation of protein was observed pelleted following centrifugation. Antigen detection among the pooled E/S media previously heated at increasing temperatures showed relatively stable antigen detection, (Fig. 4), with a significant difference relative to the 25 °C reference only observed for the female E/S media 95 °C heated samples.

### Effect of pepsin digestion and TCEP disulfide bond reduction of cultured heartworm ES media on antigen detection

**Fig. 5:**
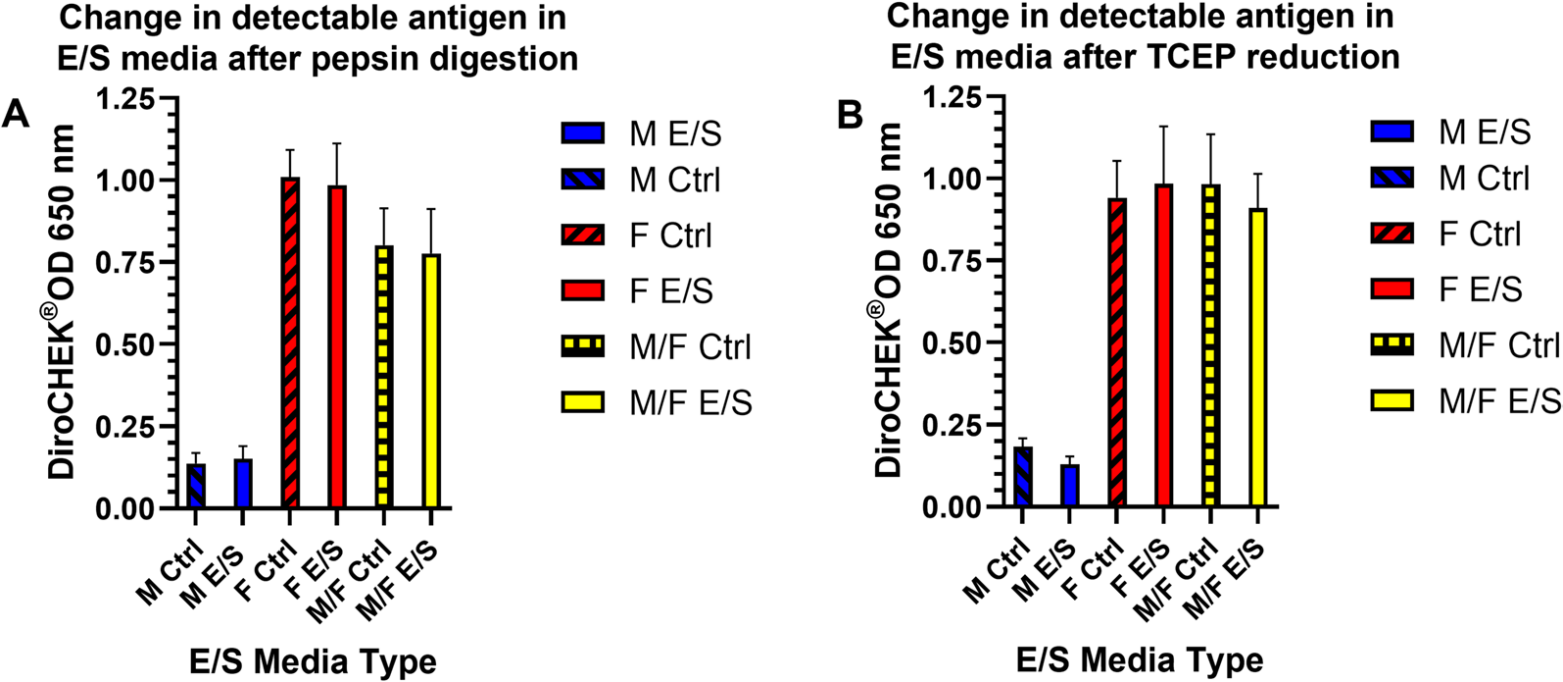
Effect of **A** pepsin digestion and **B** Tris[2-carboxyethyl] phosphine hydrochloride (TCEP) on antigen detection in pooled excretory/secretory (E/S) media from live heartworms as measured by DiroCHEK^®^ via optical density at 650 nm. No significant changes to antigen detection among these E/S media were observed

Non-specific enzymatic digestion of E/S media cultures by immobilized pepsin showed no significant effect on antigen detection as shown in Fig. 5. Similar results were seen following TCEP reduction of disulfide bonds in E/S media in that no significant changes in antigen detection were observed (Fig. 5). This suggests that the epitope is not affected by protein digestion or disulfide bond reduction, both of which should affect the conformation of the glycoprotein, supporting a linear epitope.

## Discussion

This study was designed to confirm the mechanism of action of heat treatment (heat ICD) shown to improve sensitivity of heartworm antigen tests as a result of dissociation of antibody from antigen bound in immune complexes. Additional questions concerning the effect of the heat on heartworm antigen are also addressed by testing crude antigen in the form of excretory/secretory products from live heartworms. Using this crude antigen, referred to as ES media, additional information regarding the linear versus conformational nature of the target epitope recognized by test reagents was generated.

The hypothesis that increased heat improves heartworm antigen detection resulting from heat-induced changes to the antigen-specific antibody was tested by heating isolated canine IgG alone in the absence of heartworm antigen. Changes to the canine IgG, following heating of individual aliquots of four antibody solutions (Ab1–4) at six temperatures, were measured by protein quantification and functional ability of antibody to bind to free antigen, as measured by antigen detection/OD, when mixed with nonheated antigen-positive serum.

Results shown in Fig. 1, suggest that increasing heat has a significant effect on soluble total protein in antibody aliquots. Antibody solutions heated at 65 °C showed no significant difference in protein concentration compared to the 25 °C control, which represents the native isolated canine IgG. A significant increase in protein concentration occurred in antibody solutions heated at 75 °C, suggesting fragmentation of antibody domains and concordant increase in free soluble protein. Significant decreases in protein concentration were then observed for aliquots heated at 85 °C, 95 °C and 104 °C, all relative to the 25 °C control temperature, suggesting increased denaturation and aggregation of the antibody out of solution.

The significant changes seen in Fig. 1 can be extended to Fig. 2, which demonstrates the interfering effect or lack thereof for these same heated antibody solutions on nonheated antigen-positive serum (Ag1–4). In Fig. 1, protein levels are not significantly changed between the 25 °C control and 65 °C and correspond to the depressed antigen detection observed following mixing of 25 °C and 65 °C heated aliquots with Ag1–4, as shown in Fig. 2. These results indicate viable antibody binding and function and formation of immune complexes. Conversely, for the antibody aliquots heated at temperatures of 75 °C, 85 °C, 95 °C and 104 °C in which significant changes in soluble protein were observed, when mixed with positive serum Ag1–4 additionally showed a restoration of antigen detection/OD to levels at or above the OD of the initial controls (Ag1–4/PBS). These results suggest that initial changes to the Fab portion of the canine IgG antibody, which impact the ability to bind the targeted epitope of the heartworm antigen, initially occur at temperatures between 65 °C and 75 °C. Given that this experiment only used heated antibody solutions (adjusted back to original volume post-heat) and nonheated antigen-positive serum, any effect observed was due to a change in the antibody alone. Previous reported analysis on heat stability of murine IgG 2b concluded irreversible denaturation of the individually isolated Fab and Fc domains occurs at 61 °C and 71 °C, respectively [22]. Based on the temperature intervals evaluated in our study using intact total IgG, the change to the canine IgG Fab domain initially occurs at temperatures between 65 °C and 75 °C. These data suggest that although heat ICD at 104 °C is used for ICD pre-treatment of serum or plasma for heartworm testing, lower temperatures may be sufficient for some degree of immune complex dissociation [16]. The use of lower temperature would need to be further investigated and may be useful when applying the technique to antigen detection of other infectious or parasitic agents.

The effect of heat on the target heartworm antigen was evaluated using culture supernatants from live heartworms; using this source we effectively obtained the antigen among other excretory and secretory products. By using this E/S media, the target antigen is free from host antibody, thus allowing experiments on the antigen in vitro. This media however likely contains additional lower molecular weight proteins with the targeted epitope, which are normally filtered from the blood by the kidneys and expelled in the urine [4]. This would explain the observed detectable antigen in urine from experimentally infected and pet dogs recently reported [23]. In Fig. 3, heating of the ES media showed a visually non-significant decrease in detected protein concentrations for all heating temperatures; the only significant change occurred in all three heartworm culture groups at 104 °C. Interestingly, in a recent paper looking at antigen detection in urine, antigen detection was reduced following heat ICD of urine [23] versus antigen detection in non-heated urine. The authors suggested this reduction may be due to reduced antigen stability in urine. However, in context of the results reported here, those data from experimental and in-clinic animals likely confirm our data suggesting lower molecular weight glycoprotein antigens removed from circulation and excreted in urine [4] may be less heat stable at 104 °C, which may explain in part the significant changes observed in Fig. 3. It would be interesting to see if a reduction in detectable antigenuria would occur on those same urine samples [23] when re-evaluated at the lower temperatures examined here for serum. Although no visible protein was precipitated in the heated ES media aliquots, measured protein decreased. However, these values in Fig. 3 may not accurately reflect changes in concentration of the target antigen in blood circulation because of the crude nature of the E/S media containing all released excretory/secretory products, including those normally filtered from circulation. In fact, the minor change in protein levels among the crude ES media antigens did not match any similar reduction in the target heartworm antigen, as indicated in Fig. 4, mostly showing no significant changes in detectable antigen with the exception of 95 °C for the female ES media. The visual trend of a general decrease in protein concentration suggests that some proteins present are being denatured and aggregated out of solution with heat, as would be expected and supported by data in Fig. 4 for temperatures ≥ 85 °C. These data suggest that the targeted epitope on the ES antigen is not significantly affected by heat. Data which should likewise extend to heartworm antigen in positive serum in that post-heat ICD antigen remains soluble in solution and does not significantly aggregate with host antibody. Heartworm antigen data for these same heated aliquots of ES media suggest that the heating has minimal effect on the targeted epitope of the antigen and additional cryptic epitopes are not available and are suggestive of a linear nature of the epitope. This possibility is further supported by antigen testing following non-specific enzymatic digestion of the ES media by pepsin, as shown in Fig. 5, which should affect available peptide bonds of any amino acid component of the target epitope if present.

Additional support for a linear epitope is evidenced following TCEP reduction, which should elucidate involvement of disulfide bonding between protein chains possibly contributing to a conformational epitope, shown in Fig. 5. No significant differences in observed antigen detection following TCEP reduction of E/S media were observed. Weil et al. [4] reported that a near complete loss of antigen activity in the two primary glycoprotein target antigens occurred following periodate treatment [4] known to cleave carbon–carbon bonds of many possible 1,2-difunctionalized alkanes present in glycoproteins [24]. Whether 1,2-difunctionalized alkanes or the potential variety of functional groups is involved directly in the linear epitope is unknown. Altogether these data also suggest the presence of a heat stable linear epitope present on the targeted heartworm antigen.

## Conclusions

These experimental data support the proposed mechanism of dissociation of immune complexes via denaturation and aggregation of canine IgG and improved detection of the diagnostic epitope following heat treatment. These experiments using heat, enzymatic and chemical treatments suggest no significant changes occur in the targeted antigen and that a linear epitope is probably involved as antigen detection is seemingly not affected by potential changes in protein confirmation following heating. Lower temperatures may potentially be useful for heat ICD for heartworm antigen detection and may allow for additional improved sensitivity for male heartworms or possibly immature heartworm infections currently undetectable even after heat ICD at 104°C [9, 16]. Additionally, the data reported here using lower temperatures for heat-ICD may be relevant for improving the sensitivity of antigen diagnostic tests targeting other parasitic and vector-borne diseases where sensitive antigen detection is impacted by immune complexing or potentially for scenarios where higher temperatures used for heat ICD have an undesired impact on test specificity.

## Data Availability

All data generated or analyzed during this study are included in the published article.

## Abbreviations

ICD: Immune complex dissociation
PBS: Phosphate-buffered saline
OD: Optical density
kDa: Kilodalton
MWCO: Molecular weight cut-off
ES: Excretory/secretory
IgG: Immunoglobulin G
ELISA: Enzyme-linked immunosorbent assay
TCEP: Tris[2-carboxyethyl] phosphine hydrochloride
RPMI 1640: Roswell Park Memorial Institute 1640 Medium
HEPES: 4-(2-Hydroxyethyl)-1-piperazineethanesulfonic acid

## References

[1] Karavodin LM, Ash R (1980). Circulating immune complexes in experimental filariasis. Clin Exp Immunol.

[2] Weil GJ (1987). *Dirofilaria immitis*: identification and partial characterization of parasite antigens in the serum of infected dogs. Exp Parasitol.

[3] Weil GJ, Malane MS, Powers KG (1984). Detection of circulating parasite antigens in canine dirofilariasis by counterimmunoelectrophoresis. Amer J Trop Med Hyg.

[4] Weil G, Malane M, Powers K, Blair LS (1985). Monoclonal antibodies to parasite antigens found in the serum of *Dirofilaria immitis*-infected dogs. J Immun.

[5] Wheat LJ, Walsh TJ (2008). Diagnosis of invasive aspergillosis by galactomannan antigenemia detection using an enzyme immunoassay. Euro J Clin Microbio Infect Dis.

[6] Swartzentruber S, LeMonte A, Witt J, Fuller D, Davis T, Hage C (2009). Improved detection of *Histoplasma* antigenemia following dissociation of immune complexes. Clin Vacc Immunol.

[7] Beall MJ, Arguello-Marin A, Drexel J, Liu J, Chandrashekar R, Alleman AR (2017). Validation of immune complex dissociation methods for use with heartworm antigen tests. Parasit Vectors.

[8] Little S, Saleh M, Wohltjen M, Nagamori Y (2018). Prime detection of *Dirofilaria immitis*: understanding the influence of blocked antigen on heartworm test performance. Parasit Vectors.

[9] Gruntmeir JM, Thompson NM, Long MT, Blagburn BL, Walden HD (2021). Detection of heartworm antigen without cross-reactivity to helminths and protozoa following heat treatment of canine serum. Parasit Vectors.

[10] Krenacs L, Krenacs T, Stelkovics E, Raffeld M (2010). Heat-induced antigen retrieval for immunohistochemical reactions in routinely processed paraffin sections. Meth Mol Biol.

[11] Little SE, Munzing C, Heise SR, Allen K, Starkey LA, Johnson EM (2014). Pre-treatment with heat facilitates detection of antigen of *Dirofilaria immitis* in canine samples. Vet Parasitol.

[12] Little SE, Raymond MR, Thomas JE, Gruntmeir J, Hostetler JA, Meinkoth JH (2014). Heat treatment prior to testing allows detection of antigen of *Dirofilaria immitis* in feline serum. Parasit Vectors.

[13] Velasquez L, Blagburn BL, Duncan-Decoq R, Johnson EM, Allen KE, Meinkoth J (2014). Increased prevalence of *Dirofilaria immitis* antigen in canine samples after heat treatment. Vet Parasitol.

[14] Drake J, Gruntmeir J, Merritt H, Allen L, Little SE (2015). False negative antigen tests in dogs infected with heartworm and placed on macrocyclic lactone preventives. Parasit Vectors.

[15] Gruntmeir JM, Adolph CB, Thomas JE, Reichard MV, Blagburn BL, Little SE (2017). Increased detection of *Dirofilaria immitis* antigen in cats after heat pretreatment of samples. J Fel Med Surg.

[16] Gruntmeir JM, Long MT, Blagburn BL, Walden HS (2020). Canine heartworm and heat treatment: An evaluation using a well based enzyme-linked immunosorbent assay (ELISA) and canine sera with confirmed heartworm infection status. Vet Parasit.

[17] Carmichael J, McCall S, DiCosty U, Mansour A, Roycroft L (2017). Evaluation of *Dirofilaria immitis* antigen detection comparing heated and unheated serum in dogs with experimental heartworm infections. Parasit Vectors.

[18] Ames MK, VanVranken P, Evans C, Atkins CE (2020). Non-Arsenical heartworm adulticidal therapy using topical moxidectin-imidacloprid and doxycycline: A prospective case series. Vet Parasitol.

[19] AHS: Current Canine Guidelines for the Prevention, Diagnosis, and Management of Heartworm (*Dirofilaria immitis*) Infection in Dogs. 2020:35.

[20] Kotani T, Powers KG (1982). Developmental stages of *Dirofilaria immitis* in the dog. Am J Vet Res.

[21] Lichtenfels JR, Pilitt PA, Kotani T, Powers KG (1985). Morphogenesis of developmental stages of *Dirofilaria immitis* (Nematoda) in the dog. Proc Helminthol Soc Wash.

[22] Akazawa-Ogawa Y, Nagai H, Hagihara Y (2018). Heat denaturation of the antibody, a multi-domain protein. Biophys Rev.

[23] Brown AC, Saleh MN, Fudge JM, Nabity MB, Verocai GG (2022). Evaluation of urine for *Dirofilaria immitis* antigen detection in dogs. Res Sq.

[24] Dryhurst G (2015). Periodate oxidation of diol and other functional groups: analytical and structural applications.

